# Job stressors and burnout among clinical nurses: a moderated mediation model of need for recovery and career calling

**DOI:** 10.1186/s12912-023-01524-1

**Published:** 2023-10-18

**Authors:** Tingting Jin, Yaoying Zhou, Leigang Zhang

**Affiliations:** 1 Surgery Intensive Care Unit, Shaoxing People’s Hospital, Shaoxing, Zhejiang China; 2 Nursing Department, Shaoxing People’s Hospital, Shaoxing, Zhejiang China; 3 College of Teacher Education, Shaoxing University, Shaoxing, Zhejiang China

**Keywords:** Job stressors, Need for recovery, Career calling, Burnout

## Abstract

**Background:**

Burnout is a major concern in healthcare professions. Although theory and empirical evidence support the relationship between job stressors and burnout, the question remains how and when the job stressors are related to burnout. Based on conservation of resources theory and effort recovery model, the current study aimed to provide a deeper understanding of the effect of job stressors on burnout by investigating the mediating role of need for recovery and the moderating role of career calling.

**Methods:**

A cross-sectional online survey was conducted among 709 nurses enrolled from eight public hospitals in China. The Work Stressors Scale, Psychological Detachment Scale, Brief Calling Scale, and Maslach Burnout Inventory were used to collect data. Hierarchical regression analysis with bootstrapping procedure was performed to test the proposed model.

**Results:**

The results showed that need for recovery mediated the job stressors-burnout relationship, and that high career calling buffered against the relationships between job stressors and need for recovery and burnout. Furthermore, the result revealed a moderated mediation model that career calling buffered the indirect effect of job stressors on burnout through need for recovery.

**Conclusions:**

Our findings suggest that environmental demands and personal resource are important antecedents of nurses’ burnout. Career calling as personal resources can serve as a protective factor that guards against burnout. Thus, nursing managers can reduce nurse burnout by focusing on effective strategies related to recovery experiences, as well as investing in training career calling.

## Introduction

Nurses are known to be at high risk of job stress and burnout. Recent statistics indicate that 20–40% of nurses report experiencing burnout [1, 2]. Burnout not only deteriorates nurses’ work performance but also adversely influences their health and well-being [3]. To mitigate the negative effects of burnout, it is crucial to gain more knowledge about the antecedents and process of burnout, and to identify moderators that could help. The wide variety of work-related stressors, like high workload and time constraints, are the most important factors in a nursing context. Previous research reported that particularly high job demands are important antecedents of increased burnout as a long-term consequence [4, 5]. Nurses experience burnout when their workloads are increased by systemic problems, such as irregular hours, shift-work, and high emotional demands [6, 7].

Although job demands may induce a need for recovery, resulting in psychological fatigue and emotional exhaustion [8], not all individuals who are faced with job demands suffer from burnout. For instance, despite reports of high job demands, some nurses may still work enthusiastically and be immersed in their work as they believe doing so is important and meaningful, even though they simultaneously recognize that doing so may leave them feeling exhausted [9]. In the stress literature, career calling is one of the most salient constructs affecting how individuals respond to work-related stressors. From the conservation of resources theory perspective, career calling is regarded as a type of personal resource that may help individuals resist the detrimental effects of work stressors [10]. Only a few studies have investigated the moderating role of career calling in the relationship between job stressors and burnout. Thus, the current study was designed to examine the relationships between job stressors, need for recovery, career calling, and burnout among nursing staff.

## Background

### Job stressors and burnout among nurses

Burnout is a syndrome that combines emotional exhaustion, depersonalization, and reduced personal accomplishment [11]. Emotional exhaustion, a central component of burnout, refers to feelings of being overextended and depleted of emotional and physical resources. Depersonalization involves a negative or overly detached attitude to others. Lastly, reduced personal accomplishment is described as a decline of feelings of competence and successful achievement in one’s work. International studies indicate that nurses may be at greater risk of burnout compared with other professional group, as they are working in settings with the combination of high job demands and low job control [12].

Job demands are prevalent job stressors among nurses. Generally, nursing job demands are determined by time spent on patient care, nursing activities, and the skills needed to care for the patient [13]. Even the experienced nurses perceive the increasingly complex working conditions similarly challenging to cope with. Increased job demands can negatively affect nurses physically and psychologically. The link between job stressors and burnout can be viewed through the lens of conservation of resources (COR) theory [12]. COR theory hypothesizes that individuals tend to obtain, maintain, and protect the personal characteristics, conditions, and energies that enable them to cope with job demands [14]. According to the COR theory, the physical, cognitive or emotional efforts needed to cope with high job demands are common sources of job stressors. If the demands of a stressful job exceed their physical or mental resources, and deplete personal resources at work, people may experience high burnout [15, 16]. Therefore, consistent with the aforementioned empirical evidence and premised on the COR theory, we aimed at replicating this relationship between high job stressors and increased burnout.

### Job stressors and need for recovery

For both physical and mentally demanding activities, when fatigue builds up, people want to stop thinking about activities and have a break. This sense of urgency refers to the need for recovery, which is defined as the degree to which an employee needs to recuperate both physically and mentally from the effort spent on doing his/her work tasks [17]. Need for recovery is linked to the expectation that such a break is inevitable in order to be able to continue with the present demands or to accept future demands. Typically, the recovery process takes place in the after-work period. Recovery experiences include pursuits that people engage in social and physical off-work activities (e.g., sports or travel), or low-effort (e.g., watching TV or going to a concert) activities [18]. In relation to health, need for recovery by itself is not a worrying concept. Nevertheless, inability to rest and recover from work is detrimental for health and well-being because of the accumulation of strain, whereas successful recovery facilitates employee flourishing. Therefore, it is assumed that need for recovery can be seen as a pre-phase of burnout and an entry point to prevent prolonged fatigue.

Extending from conservation of resources theory and effort recovery model, need for recovery is hypothesized to develop from immediate and sustained responses to stressors or demands that we are exposed to within daily work. According to COR theory, in addition to striving to save and protect resources, people endeavor to acquire extra resources to maintain health and well-being. When actual resources are lost, individuals attempt to minimize stress by taking actions to replenish energy. Therefore, recovery not only reflects the process of replenishing depleted or lost resources, but also implies that resource-loss cycles are halted. High need for recovery during non-work time implies that employees are strained due to dealing with job stressors. Complimentary to COR theory, the effort-recovery model proposes that high job demands trigger physiological and subjective load reactions that increase employee strain, with these effects being reversible via the process of recovery if these demands cease [19]. The chronic exposure to job stressors makes recovery necessary in an objective way and increases people’s subjective need for recovery, because job stressors deplete affective and energetic resources immediately during the working day. When people feel that they are not sufficiently recovered, they may feel physical and emotional depletion, subsequently they will lack the energy to cope with continuing or new demands. By contrast, when recovery from work has been optimal, employees have no feelings of work-related stress when accepting new challenges.

Empirical studies using diverse study designs and occupational groups have confirmed a positive link between job stressors and need for recovery [20, 21]. For example, a study using diverse samples, including coach drivers, public bus drivers, truck drivers, construction workers, ambulance workers, and hospital nurses, confirmed that job demands were significant predictors of need for recovery [22]. Researchers also report that psychological work demands had a greater effect on need for recovery than physical work demands in ageing seafarers [23].

### Need for recovery as a mediator

Previous research findings support assumptions that people with high need for recovery are at an increased risk of developing occupational diseases. For example, high need for recovery is considered as an early precursor for developing high blood pressure [24], cardiovascular disease [25], and musculoskeletal problems [26]. Need for recovery is furthermore strongly related to negative phenomena such as fatigue and emotional exhaustion [27]. These studies suggest that the experienced level of need for recovery is proportional to the fatigue cumulated during the working time. The higher the need for recovery, the higher the strain experienced. Need for recovery is seen as an early symptom of work-related fatigue that describes the early stages of strain process and successively translates into a long-lasting condition of energy depletion. Recovery experiences thus are critical to maintaining occupational health and well-being.

Although job demands are not necessarily negative, they may turn into job stressors when meeting such demands requires high investment of limited resources. Resource depletion puts individuals at more risk for experiencing the negative consequences of stress, followed by the inability to deal effectively with stressors and to recover from stress, which, in turn, contributes to burnout. Studies have investigated need for recovery as a process that mediates between work stressors and work outcomes [27]. For example, the need for recovery has been demonstrated to act as an intermediate stage between exposure to stressful working conditions and the development of psychosomatic health problems in the longer term [28]. Accordingly, we propose that need for recovery functions as a mediator in the relationship between job stressors and burnout. Specifically, job stressors are assumed to be positively associated with need for recovery, and high need for recovery in turn is assumed to be associated with higher burnout.

### Career calling as a moderator

Multiple definitions and operationalizations of career calling have emerged over past two decades. For the purpose of current study, we apply Dik and Duffy’s definition for calling as a “transcendent summons, experienced as originating beyond the self, to approach a particular life role in a manner oriented toward demonstrating or deriving a sense of purpose or meaningfulness and that holds other-oriented values and goals as primary sources of motivation’’ [29]. Individuals with career calling regard their work to be their purpose in life rather than just a means for financial rewards or career advancement [30]. For nurses, having a calling is particularly important. Nurses with high sense of calling can be described as nurses who are enthusiastic about nursing, attentive to their patient’s needs, more engaged and absorbed in their work, and more persistent when faced with obstacles.

Studies have shown that career calling is related to a variety of positive individual and organizational outcomes, such as higher career commitment and occupational identity [31, 32], higher job satisfaction and performance [33], as well as lower turnover and withdrawal intentions [34]. Moreover, career calling is considered as a psychological resource that predicts higher career confidence, career resilience, career adaptability, and career self-efficacy [35, 36]. Individuals with high career calling are also more capable of anticipating work problems and better able to find proactive ways to address them. For example, some scholars view career calling as a self-regulatory strategy that individuals possess and use in response to challenging work situations [37]. Higher calling is associated with more work effort, greater use of career strategies, and higher emotional regulation. Those who adopt a calling orientation tend to engage in various behavioral and psychological processes that direct their attention toward meaningful career-related activities.

Career calling has been also found to moderate the relationship between role conflict and job burnout [38]. Individuals with career calling consider job stressors as a challenge rather than a threat, thus, they put more effort into experiencing hurdles and using effective coping strategies. These results corroborate the importance of enhancing career calling to reduce nurses’ burnout. Guided by conservation of resources theory, we suggest that calling can be seen as a personal resource that aids individuals in resisting the adverse outcomes of job stressors. Specifically, career calling as a moderator can attenuate the positive effects of job stressors on need for recovery and burnout. This study also predicts a moderated mediation effect, with career calling expected to serve as a buffer of the indirect effect of job stressors on nurses’ burnout through need for recovery. Since those high in career calling are more likely to immerse themselves in them, they are less likely to distance oneself from the job, thus they tend to experience low need for recovery and burnout.

### The present study: aims and hypotheses

**Fig. 1:**
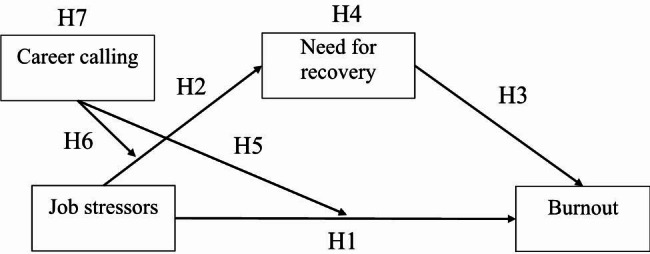
Moderated mediation model

Drawing on conservation of resources theory and effort-recovery model, this study aims to explore why and when job stressors are associated with burnout. Based on the literature review, we propose a moderated mediation model where the indirect effect of job stressors on burnout via need for recovery may depend on the level of career calling. The hypothesized moderated mediation model is presented in Fig. 1, comprising the following seven hypotheses:

#### Hypothesis 1

Job stressors are positively related to burnout.

#### Hypothesis 2

Job stressors are positively related to need for recovery.

#### Hypothesis 3

Need for recovery is positively related to burnout.

#### Hypothesis 4

Need for recovery mediates the relationship between job stressors and burnout.

#### Hypothesis 5

Career calling will weaken the positive relationship between job stressors and burnout.

#### Hypothesis 6

Career calling will weaken the positive relationship between job stressors and need for recovery.

#### Hypothesis 7

Career calling will moderate the strength of the mediated relationship between job stressors and burnout via need for recovery, such that the mediated relationship will be weaker for those who experience high career calling.

## Methods

### Sample and procedure

This study employed a cross-sectional, correlational survey design with an online survey. Data were collected between May and October 2020 from a convenience sample of registered nurses recruited from eight public hospitals in China. After obtaining the permission and cooperation of the nursing departments of the hospitals, a sample of 760 registered nurses were recruited. Volunteer nurses who completed the informed consent form could follow a link to the online survey. The survey was approximately 15 min in length. To encourage nurses to participate in this study voluntarily, they were paid ¥ 5.0 for their participation.

Of these 760 participants, 51 had missing data on job stressor, need for recovery, calling or burnout and were removed from further analysis leaving 709 cases. According to Bentler and Chou [39], the sample size should be more than 10 times the observed variables. Thus, a sample size of 709 met the requirement for further analysis. Of the participants, 97.6% were female. The age of the participants ranged from 25 up to 53 years, with an average of 39.27 years. The largest group was between 31 and 40 years old (44.4%), followed by those 41–50 years old (35.4%), those above 51 yeas old (10.9%), and those below 30 years old (9.3%). In terms of educational attainment, 2.70% had some diploma degree, 88.6% had a bachelor’s degree, and 0.4% had a master’s degree. The characteristics of the study sample are summarized in Table 1.

**Table 1:** Demographic variables of nurses (N = 709)

	Frequency (%)
**Age**	
Below 30	66 (9.3%)
31–39	315 (44.4%)
40–50	251 (35.4%)
Above 51	77 (10.9%)
**Gender**	
Male	17 (2.4%)
Female	692 (97.6%)
**Marital status**	
Single	157 (22.1%)
Married	544 (76.7%)
Divorced	8 (1.1%)
**Level of education**	
Diploma degree	78 (11.0%)
Bachelor’s degree	628 (88.6%)
Master’s degree	3 (0.4%)
**Years of work experience**	
Less than 5 years	212 (29.9%)
6–15 years	328 (46.2%)
16–25 years	89 (12.6%)
More than 25 years	80 (11.3%)

### Measurement

The measurements were implemented in the following order: Demographic Questionnaire, Work Stressors Scale (WSS), Need for Recovery Scale (NRS), Brief Calling Scale (BCS), and Maslach Burnout Inventory (MBI). Except for the demographic questionnaire, all of the items were answered on a 5-point Likert-type scale(ranging from 1 = strongly disagree to 5 = strongly agree). Scale scores were computed by averaging across scale items.

### Demographic questionnaire

Demographic questionnaire was used to collect information on participants’ age, gender, marital status, level of education, and years of work experience.

### Job demand scale (JDS)

The JDS developed by Karasek et al. is a 3-item scale that measures an individual’s perceptions of physical demands [40]. A sample item is “I have to work very intensely in my job.” Higher scores indicate higher level of physical demands. In the original study, Cronbach’s alpha coefficient was 0.86 and 0.79 for men and women respectively, demonstrating good reliability. In current study, the Cronbach’s α was 0.84.

### Need for recovery scale (NRS)

The eleven-item need for recovery scale developed by Sluiter et al. was used to assess the short-term effects of a day of work [41]. A sample item is “After a working day I am often too tired to start other activities”. Higher scores indicate worse recovery from work. In their instrument development study, Sluiter et al found an internal consistency of α = 0.89. In current study, the Cronbach’s α was 0.77 and the confirmative factor analysis result showed that the single-dimensional model was a good fit (χ^2^/df = 2.18, RMSEA = 0.05, GFI = 0.99, TLI = 0.98, CFI = 0.97).

### Brief calling scale (BCS)

The two-item presence of calling subscale from Dik’s BCS was used to assess the degree to which participants experienced the presence of a calling [42]. The two items include, “ I have a calling to a particular kind of work”, and “I have a good understanding of my calling as it applies to my career”. In the original study, the scale was found to have good internal consistency (α = 0.90). In current study, the Cronbach’s α was 0.76.

### Maslach burnout inventory (MBI)

The MBI developed by Maslach and Jackson was used to assess burnout [43]. The scale consists of 15 items, including three subscales: emotional exhaustion (5 items; e.g., “I feel frustrated by my job”), cynicism (4 items; e.g., “I have become less interested in my work since I started this job”), and decreased personal accomplishment (6 items; e.g., “I feel exhilarated when I accomplish something at work”). Items measuring decreased personal accomplishment were reverse coded so that higher scores indicate higher levels of burnout. The Cronbach’s alphas of internal consistency reliability for the emotional exhaustion, cynicism, and decreased personal accomplishment were respectively 0.89, 0.72, and 0.74 in the instrument development study and 0.75, 0.68, and 0.79 in the current study. The Cronbach’s alphas of the current study was 0.81 for the general score of MBI. The confirmatory factor analysis results in this study showed that the three-dimensional model was a good fit (χ2/df = 2.92, RMSEA = 0.06, GFI = 0.97, TLI = 0.95, CFI = 0.95).

### Ethical considerations

Ethical approval was granted by the institutional review board of Shaoxing University, and permission to collect data was granted by each hospital. Before conducting the questionnaire survey, all participants received an introduction letter inviting them to participate and informing them about the purpose of the study and procedure to follow. Written informed consent was obtained during the initial stage of the study. Participants were assured of confidentiality and anonymity, they could withdraw from the study at any moment and for any reason.

### Analytic procedure

Data were analysed using SPSS version 21.0 and Amos version 20.0. The means, standard deviations, Cronbach’s α and correlations among variables were calculated. Hierarchical multiple regression analysis with bootstrapping approach embedded in the PROCESS macro was used to test our hypotheses. Specifically, first, a multiple regression analysis was conducted to test the direct and the indirect effect of job stressors on burnout via need for recovery. In testing mediation, we used 2,000 bootstrapping samples to obtain the 95% bias-corrected confidence intervals (CIs). Mediation occurs if the zero is not included in the 95% CIs. Second, the moderating role of career calling in the effects of job stressors on need for recovery and burnout was examined using hierarchical linear regression analyses followed by the simple slope test suggested by Aiken and West [44]. Finally, to test the moderated mediation hypotheses, we utilized Preacher’s approach [45]. It estimates the conditional indirect effects at low, intermediate, and high levels of the moderator. The index of moderated mediation was also calculated. This index should be different from zero in order to support our hypotheses.

## Results

### Descriptive statistics

Means, standard deviations, Cronbach’s α, and correlations are displayed in Table 2. Job stressors were significantly and positively associated with need for recovery (*r* = 0.62, *p* < 0.01) and burnout (*r* = 0.38, *p* < 0.01), and negatively associated with career calling (*r* = -0.35, *p* < 0.01). Need for recovery was significantly and positively associated with burnout (*r* = 0.49, *p* < 0.01), and negatively associated with career calling (*r* = -0.39, *p* < 0.01). Career calling was significantly and negatively associated with burnout (*r* = -0.42, *p* < 0.01). These significant relations support the testing of mediation and moderation analyses.

**Table 2:** Means, standard deviations, correlations, and Cronbach’s α

Variable	M ± SD	1	2	3	4
1. Job stressors	3.45 ± 0.73	(0.84)			
2. Need for recovery	3.35 ± 0.56	0.62^**^	(0.77)		
3. Career calling	3.20 ± 0.93	−0.35^**^	−0.39^**^	(0.76)	
4. Burnout	3.79 ± 0.48	0.38^**^	0.49^**^	−0.42^**^	(0.81)

Note. Cronbach’s as are presented in parentheses along the diagonal

^*^ *p < 0.05.* ^**^ *p < 0.01.*

### Testing main and mediation effects

Table 3 presents the results of the hierarchical regression analyses. Of the five control variables tested, only gender (*β* = -0.11, *p* < 0.05) and years of work experience (*β* = -0.12, *p* < 0.01) were negatively related to burnout (Model 3). In Models 1 and 3, we included the control variables and job stressors to test the main effect. The results showed that job stressors were positively related to burnout (*β* = 0.38, *p* < 0.01) and need for recovery (*β* = 0.62, *p* < 0.01). Hence, Hypotheses 1 and 2 were supported. As shown in Model 4, when need for recovery was added to the model, need for recovery was significantly and positively related to burnout (*β* = 0.40, *p* < 0.01), while the effect of job stressors on burnout dropped from 0.38 to 0.14, but was still significantly, indicating a partial mediation. Thus, hypothesis 4 was supported. To further analyze indirect effect, we calculated 95% confidence intervals (CIs) based on bias-corrected bootstrapping analyses with 2,000 samples. Specifically, Model 4 was used, where job stressors was the predictor, burnout was the outcome, and need for recovery was the mediator. Bootstrapping result demonstrated a significant indirect effect of the job stressors on burnout through need for recovery (indirect effect = 0.15, *SE* = 0.03, *p* < 0.01, 95% CI = [0.11, 0.20]). The direct effect of job stressors on burnout was also significant (direct effect = 0.09, *SE* = 0.03, *p* < 0.01, 95%CI = [0.04, 0.14]). Taken together, need for recovery partially mediated the relationship between job stressors and burnout.

**Table 3:** Results of hierarchical regression analyses

Variable	**Need for recovery**				Burnout					
	Model 1		Model 2		Model 3		Model 4		Model 5	
	*β*	*SE*	*β*	*SE*	*β*	*SE*	*β*	*SE*	*β*	*SE*
Control variables										
Age	0.02	0.03	0.01	0.03	-0.11^*^	0.04	-0.11^*^	0.04	-0.09^*^	0.03
Gender	-0.01	0.03	-0.01	0.03	-0.06	0.04	-0.05	0.03	-0.06	0.03
Marital status	-0.01	0.03	-0.02	0.03	-0.06	0.04	-0.06	0.04	-0.07	0.04
Level of education	0.02	0.04	0.01	0.03	0.01	0.04	0.01	0.03	-0.01	10.03
Years of work experience	-0.03	0.03	-0.02	0.02	-0.12^*^	0.03	-0.11^*^	0.04	-0.10^*^	0.03
Main/indirect effect										
Job stressors	0.62^**^	0.03	0.48^**^	0.03	0.38^**^	0.04	0.14^**^	0.04	0.21^**^	0.04
Need of recovery							0.40^**^	0.04		
Career calling			-0.22^**^	0.03					-0.34^**^	0.02
Interaction										
Job stressors × Career calling			0.14^**^	0.02					0.17^**^	0.03
R^2^	0.36		0.41		0.16		0.26		0.27	
⊿R^2^							0.10			
F	80.30		71.55		26.94		41.69		27.93	

### Testing moderation and moderated mediation effects

**Fig. 2:**
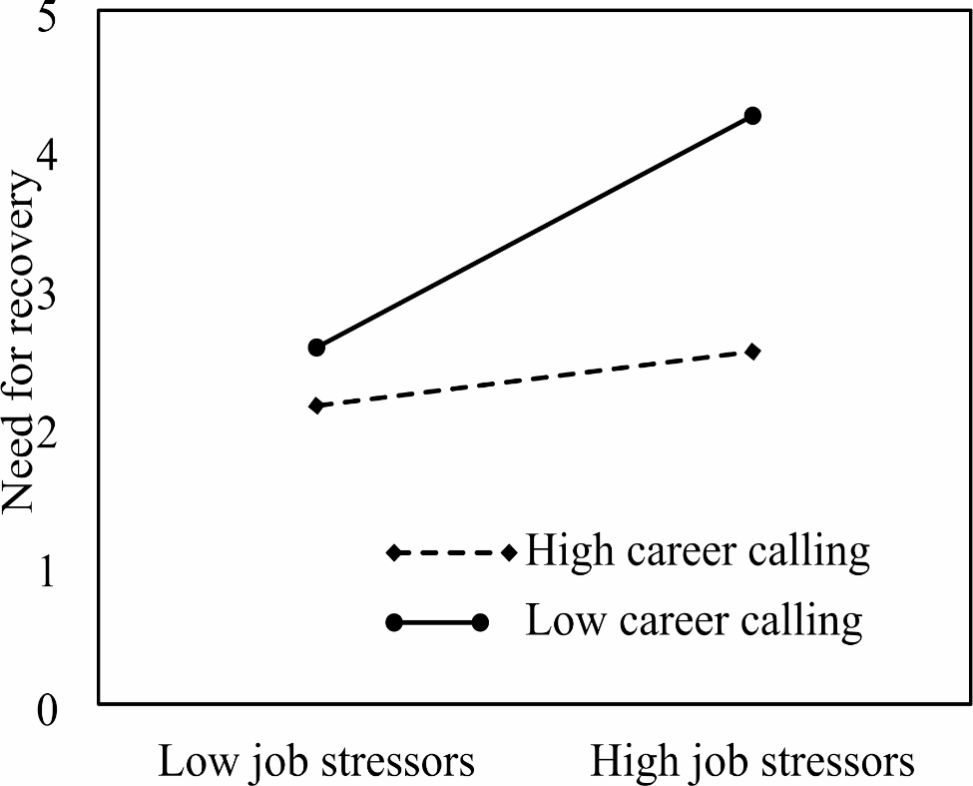
Interaction between job stressors and career calling on need for recovery

**Fig. 3:**
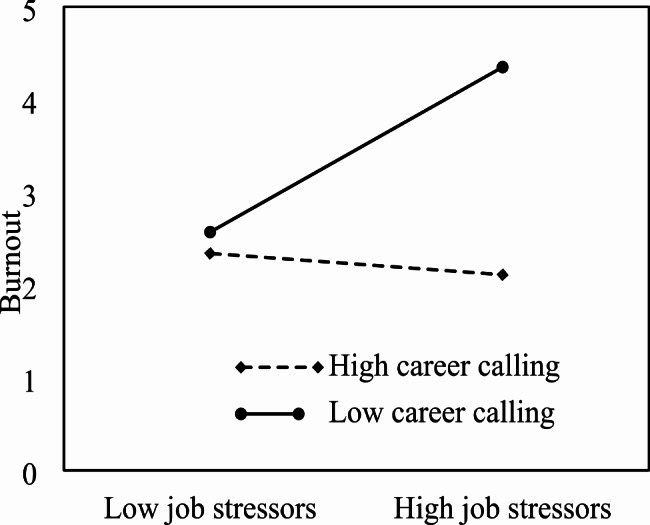
Interaction between job stressors and career calling on burnout

Hypotheses 5 and 6 concerned the moderating role of career calling in the relationship between job stressors and need for recovery and burnout. The regression analyses shown in Table 3 (Models 2 and 5) revealed that the interaction term of job stressors and career calling was positively related to need for recovery (*β* = 0.14, *p* < 0.01) and burnout (*β* = 0.17, *p* < 0.01), respectively. Following the methods recommended by Aiken and West, we calculated the simple slopes of the interaction effects one standard deviation below and above the mean to examine the nature of the significant interactions, and plotted the interactions in Figs. 2 and 3. The simple slopes analyses indicated that job stressors were not related to need for recovery for individuals high in career calling (*β* = 0.11, *t* = 1.72, *p >* 0.05), but were positively related to need for recovery for those low in career calling (*β* = 0.59, *t* = 14.65, *p* < 0.001), confirming our hypothesis that high level of career calling weakens the positive relationship between job stressors and need for recovery. Similarly, the positive relationship between job stressors and burnout was not significant for those with higher career calling (*β* = 0.09, *t* = 1.12, *p >* 0.05), but was significant for those with lower career calling (*β* = 0.32, *t* = 6.92, *p* < 0.01), indicating that high level of career calling makes the relationship between job stressors and burnout nonsignificant. This means that job stressors are only positively related to burnout under condition of low career calling. In other words, high level of career calling may buffer the effect of job stressors on need for recovery and burnout. Taken together, these findings supported Hypotheses 5 and 6. Having a higher career calling moderated the detrimental effect of job stressors, suggesting a protective effect for nurses with a stronger career calling.

Finally, we hypothesized that career calling would moderate the indirect effect of job stressors on burnout through need for recover. We tested the conditional indirect effects with Model 8 in the PROCESS bootstrapping approach provided by Hayes. It estimated the conditional indirect effect of job stressors on burnout via need for recovery at high, intermediate, and low levels of career calling. Table 4 presents the results of moderated mediation analysis. The results demonstrated that the conditional indirect effect of job stressors was stronger and significant in the low (indirect effect = 0.12, SE = 0.02, 95% CI = [0.07, 0.17]) and intermediate condition (indirect effect = 0.08, SE = 0.03, 95% CI=[0.03, 0.13]), but was not significant in the high career calling condition (indirect effect = 0.05, SE = 0.02, 95% CI=[-0.02, 0.07]). Thus, Hypothesis 7 was supported, indicating that need for recovery would mediate the relationship between job stressors and burnout, but only at lower level of career calling.

**Table 4:** Bootstrap results for conditional indirect effect of job stressors on burnout by calling

	Indirect effect	Boot SE	LL 95% CI	UL 95% CI
Low career calling (−1 SD)	0.12	0.02	0.07	0.17
Average(0)	0.08	0.03	0.03	0.13
High career calling (+ 1 SD)	0.05	0.02	−0.02	0.07
Index of moderated mediation	−0.03	0.06	−0.06	−0.01

## Discussion

The present study attempted to explain how nurses’ perceived job stressors lead to burnout and whether this relationship can be mitigated by career calling. Not surprisingly, job stressors were associated with higher need for recovery and burnout, thus supporting Hypothesis 1 and 2. Our findings are consistent with previous research and theories [46]. In the COR model, job strains are defined as aspects of the job that require sustained physical and mental efforts and are therefore associated with psychological costs. Job demands lead to stress because resources such as time and energy are lost, which can lead to health complaints and burnout. Furthermore, our study provides an initial indication that need for recovery is a possible explanation for the relationship between job stressors and burnout. Job stressors induce high need for recovery, which, in turn, has a positive effect on burnout. High need for recovery during non-work time implies that people are strained due to dealing with job demands; otherwise recovery would not be necessary. Findings highlight the fact that awareness and understanding of the need for recovery could be a primary focus of prevention of burnout among nurses, because need for recovery from work is regarded as an early stage of a long-term strain process. However, our study shows that need for recovery acts as a partial rather than full mediator, which indicates that also other processes may explain the relationship between job stressors and burnout.

We found a significant and negative relationship between career calling and burnout, such that individuals with a strong sense of career calling are not easily ‘triggered’ in periods of high workload, and might experience less burnout. This is consistent with previous finding that healthcare professionals with calling are less likely to be burned out [47]. Compared to those who approach work as a job or career, those with a calling orientation strongly identify with the work they do and believe that work is central to who they are as a person, therefore, they are more engaged in work and experience less stress and emotional exhaustion. Career calling is also an important factor in understanding what makes work meaningful. When individuals perceive their work as meaningful, they are willing to invest greater psychological and physical effort. In line with the propositions of the COR Theory, career calling, as a personal resource at work, can help individuals better cope with job-related stressors and challenges. Career calling has been found to be associated with better work well-being [48].

Furthermore, career calling was confirmed to have an important moderating role. Among nurses who possess high career calling, the relationships between job stressors and need for recovery and burnout are smaller. Also, the mediating effect of need for recovery is smaller for those with high career calling. Nurses with high career calling put forth more effort when they face challenges, so career calling as a valued personal resource can buffer the relationship between job stressors and burnout. This provides us with a more nuanced picture of responses to job stressors. According to the COR theory, high resources in a high demand environment should lead to optimal functioning, leading to a reinvestment of resources such as time and energy into the work environment. Accordingly, a nurse who has a calling may have a better understanding why she or he is caring for patients and how nursing activities are significant. From a resource perspective, career calling function as strong internal resources. Nurses with higher career calling are more easily able to protect themselves from the strains of further resource depletion because career calling provides a sense of meaningfulness and identity at work, and strengthens resilience in the face of stressful demands, whereas nurses with low career calling accrue strains that result in burnout more quickly because a deficit of meaning in work can result in burnout. Nurses with high career calling are more likely to seek new resources at work and to invest them in challenging tasks. Therefore, when facing their own difficulties, they may respond differently to these situational events by making meaning and developing greater understanding of the events. Having a deep understanding of working will allow a nurse to control her or his work and to have a more proactive attitude toward it. Given that career calling is not stable trait and fluctuates within the person, it is possible to enhance one’s sense of calling.

### Implications for nursing management

Our findings have practical implications for nursing management. First, the results highlighting the increase in job stressors as a potential antecedent of need for recovery and burnout, suggest that nursing managers should develop and implement strategies to prevent nurses from the threat of resource loss to decrease burnout. For some areas where nurses experience more threatening stress, nursing managers should consider ways to decrease workloads. However, nurses inevitably experience serious levels of strain and stressful events in workplace, it might be difficult or impossible to diminish high levels of job stressors. This study confirmed the mediating role of need for recovery in the link between job stressors and burnout. Nursing managers should encourage periods of recovery as research consistently indicates negative relationships between need for recovery and well-being. Recovery experiences, including psychological detachment, relaxation, mastery experiences and control, seems a powerful approach for nurses to reduce burnout. For example, nursing managers could devise recovery training program to help nurses effectively distance themselves from work-related issues. One way would be to build flexible work arrangements, such as flextime.

Most important, this study demonstrated that whether job stressors would be detrimental or not heavily depends on the way individuals view their work. For nurse with higher career calling, there were weaker relationships between job stressors and need for recovery and burnout. Understanding how career calling in threatening contexts provides critical insights into if and how individuals can improve their responses to stress without relying on changing the demands of a situation. Therefore, interventions aimed at improving career calling should be developed for nurses. One strategy to foster career calling is to promote nurses’ job crafting behavior. Therefore, nursing managers should make efforts to encourage job crafting behaviors, such as supporting nurses in carrying out tasks independently, giving authority and autonomy to make small changes in their job.

### Limitations and future research directions

Several additional limitations should be acknowledged and addressed in future research. First, our research recruited participants only from comprehensive hospitals, which have differences in work environments from specialized hospitals, such as salary and security systems, which may limit the generalizability of our findings. Another limitation of the study is that our sample was disproportionately female. Female may have higher need for recovery and burnout relative to male. Second, the cross-sectional nature of our data prevents us from inferring causality. Future research should use alternative research designs that strengthen causality. Longitudinal design with several data collection points can be adopted to not only establish casual links between job stressors, need for recovery, and burnout, but also explore the developmental trajectories of need for recovery and burnout. It is also notable that nurses with severe burnout symptoms may develop an attitude that current work is not their calling. To clarify the ambiguous relationship between career calling and burnout, future interventional research would help to better understand if training aimed at fostering career calling could reduce the influence of job stressors on need for recovery and burnout among nurses.

Third, researchers could examine how job stressors are appraised as a hindrance or a challenge, because these appraisals influence subsequent emotions, which in turn, can lead to employees feeling exhausted and worn out. For example, hindrance stressors tend to trigger negative emotions and a passive style of coping such as those reflected in greater burnout. Our results reveal that job stressors are positively linked to need for recovery and burnout. However, this relationships might also weaken under conditions of high challenging work demands. Future research might test the differential effects of hindering versus challenging work demands on need for recovery and burnout.

## Conclusion

Burnout in nursing is a serious issue leading to job turnover and absenteeism, but the antecedents and process of burnout are not well understood. We proposed and examined the underlying mechanism and boundary condition associated with the effect of job stressors on burnout. In line with conservation of resources theory and effort recovery model, our findings suggested that nurses working in high demanding conditions depleted a personal resource in the form of need for recovery, which would contribute to burnout. In addition, for nurses with strong sense of calling, the positive effects of job stressors on need for recovery and burnout were diminished. Therefore, organizations providing nurses with adequate rest periods should also help them discover or find a calling in nursing. Overall, the present study extended understanding of how and when job stressors are positively associated with burnout.

## Data Availability

The data that support the findings of this study are available from the corresponding author on reasonable request.
